# Modeling DNA damage-induced pneumopathy in mice: insight from danger signaling cascades

**DOI:** 10.1186/s13014-017-0865-1

**Published:** 2017-08-24

**Authors:** Florian Wirsdörfer, Verena Jendrossek

**Affiliations:** Institute of Cell Biology (Cancer Research), University Hospital Essen, University of Duisburg-Essen, Virchowstrasse 173, Essen, Germany

## Abstract

Radiation-induced pneumonitis and fibrosis represent severe and dose-limiting side effects in the radiotherapy of thorax-associated neoplasms leading to decreased quality of life or - as a consequence of treatment with suboptimal radiation doses - to fatal outcomes by local recurrence or metastatic disease. It is assumed that the initial radiation-induced damage to the resident cells triggers a multifaceted damage-signalling cascade in irradiated normal tissues including a multifactorial secretory program. The resulting pro-inflammatory and pro-angiogenic microenvironment triggers a cascade of events that can lead within weeks to a pronounced lung inflammation (pneumonitis) or after months to excessive deposition of extracellular matrix molecules and tissue scarring (pulmonary fibrosis).

The use of preclinical in vivo models of DNA damage-induced pneumopathy in genetically modified mice has helped to substantially advance our understanding of molecular mechanisms and signalling molecules that participate in the pathogenesis of radiation-induced adverse late effects in the lung. Herein, murine models of whole thorax irradiation or hemithorax irradiation nicely reproduce the pathogenesis of the human disease with respect to the time course and the clinical symptoms. Alternatively, treatment with the radiomimetic DNA damaging chemotherapeutic drug Bleomycin (BLM) has frequently been used as a surrogate model of radiation-induced lung disease. The advantage of the BLM model is that the symptoms of pneumonitis and fibrosis develop within 1 month.

Here we summarize and discuss published data about the role of danger signalling in the response of the lung tissue to DNA damage and its cross-talk with the innate and adaptive immune systems obtained in preclinical studies using immune-deficient inbred mouse strains and genetically modified mice. Interestingly we observed differences in the role of molecules involved in damage sensing (TOLL-like receptors), damage signalling (MyD88) and immune regulation (cytokines, CD73, lymphocytes) for the pathogenesis and progression of DNA damage-induced pneumopathy between the models of pneumopathy induced by whole thorax irradiation or treatment with the radiomimetic drug BLM. These findings underline the importance to pursue studies in the radiation model(s) if we are to unravel the mechanisms driving radiation-induced adverse late effects.

A better understanding of the cross-talk of danger perception and signalling with immune activation and repair mechanisms may allow a modulation of these processes to prevent or treat radiation-induced adverse effects. Vice-versa an improved knowledge of the normal tissue response to injury is also particularly important in view of the increasing interest in combining radiotherapy with immune checkpoint blockade or immunotherapies to avoid exacerbation of radiation-induced normal tissue toxicity.

## Background

Radiotherapy to the thoracic region is an integral part of standard treatment for patients suffering from thorax-associated neoplasms, e.g. breast cancer, head and neck cancer, or non-small cell lung cancer. Although technical improvements in treatment planning that increase the accuracy of dose delivery such as stereotactic radiotherapy (RT) and intensity-modulated RT (IMRT) as well as the development of particle therapy nowadays allow to better spare normal tissue, it cannot be avoided that parts of the normal lung tissue are also exposed to ionizing radiation (IR) during thoracic irradiation. The high radiosensitivity of the normal lung tissue and its low repair capacity still remain major obstacles to successful RT or combined radiochemotherapy (RCT) of thorax-associated neoplasms. Furthermore, the interest in combining RT or RCT with immunotherapy may result in new and more severe complications [1].

Exposure of the normal lung tissue to IR triggers damage and death of resident epithelial, endothelial and immune cells and results in the activation of conserved damage response programs of the lung tissue. These include up-regulated production of cytokines/chemokines and growth factors, as well as increased recruitment of immune cells, and result in enhanced capillary permeability and edema. Further degenerative changes and sloughing of alveolar epithelia and endothelia lead to hypersecretion and alveolitis [2]. If these alterations and the inflammatory response are too excessive patients may develop clinical symptoms of pneumonitis mostly at 3–12 weeks post irradiation [3, 4]. During the following intermediate phase a reduction of the alveolar exudate and reduced inflammation can be observed indicative for the resolution of pneumonitis and a beginning regeneration [5]. Progression to lung fibrosis is associated with further changes in the lung environment such as chronic inflammation, increased levels of profibrotic mediators (e.g. growth factors, TGFβ1), hypoxia, fibroblasts recruitment/activation and deposition of extracellular matrix molecules [6, 7]. This chronic fibrotic phase starts as early as 6 months post-irradiation and culminates in the development of lung fibrosis at 6 to 24 months after irradiation or even later [3, 8–10]. Radiation-induced fibrosis is radiologically visible as a “…well-defined area of volume loss, linear scarring, consolidation, and traction bronchiectasis” [11]. It is assumed that the radiologically observed manifestations of fibrosis (e.g. subpleural, bronchiolocentric) can vary depending on the radiation technique used [11].

Despite much progress in defining cellular and molecular factors that contribute to disease pathogenesis and may therefore be suited as diagnostic or prognostic biomarkers [12, 13], so far the role of danger signalling in the response of the lung tissue to DNA damage and its cross-talk with the innate and adaptive immune systems is not well defined. A better understanding of the cross-talk of danger perception and signalling with immune activation and repair mechanisms may allow a modulation of these processes to prevent or treat radiation-induced adverse effects. Vice-versa an improved knowledge of the normal tissue response to injury is also particularly important in view of the increasing interest in combining radiotherapy with immune checkpoint blockade or immunotherapies to avoid exacerbation of radiation-induced normal tissue toxicity.

### Modeling radiation-induced adverse late effects in the lung

Exposure to IR either directly elicits damage to cellular macromolecules, particularly the DNA, or acts indirectly via the generation of free radicals and reactive ions, particularly hydroxyl radicals. Induction of DNA double strand breaks (DSB) is considered as the most toxic cellular lesion induced by IR but radiation-induced radicals can also cause damage to further cellular macromolecules such as proteins or lipids culminating in a cellular stress response, permanent arrest (senescence) or cell death [14, 15]. Irradiation of normal lung tissue by thoracic or whole body irradiation damages resident lung cells, particularly endothelial cells and alveolar epithelial cells, as well as resident immune cells [3, 16, 17]. Of further interest are biological effects that are induced in non-irradiated cells after irradiation. These so-called bystander effects include induction of DNA damage/mutations, apoptosis, altered cell activity/metabolism as well as immunomodulation and thus mimic the direct effects of irradiation in cells that have not been directly exposed to irradiation. It is thought that the pathogenic role of bystander effects in promoting radiation-induced pneumopathy may be as severe as the direct effects of irradiation [18].

**Fig. 1:**
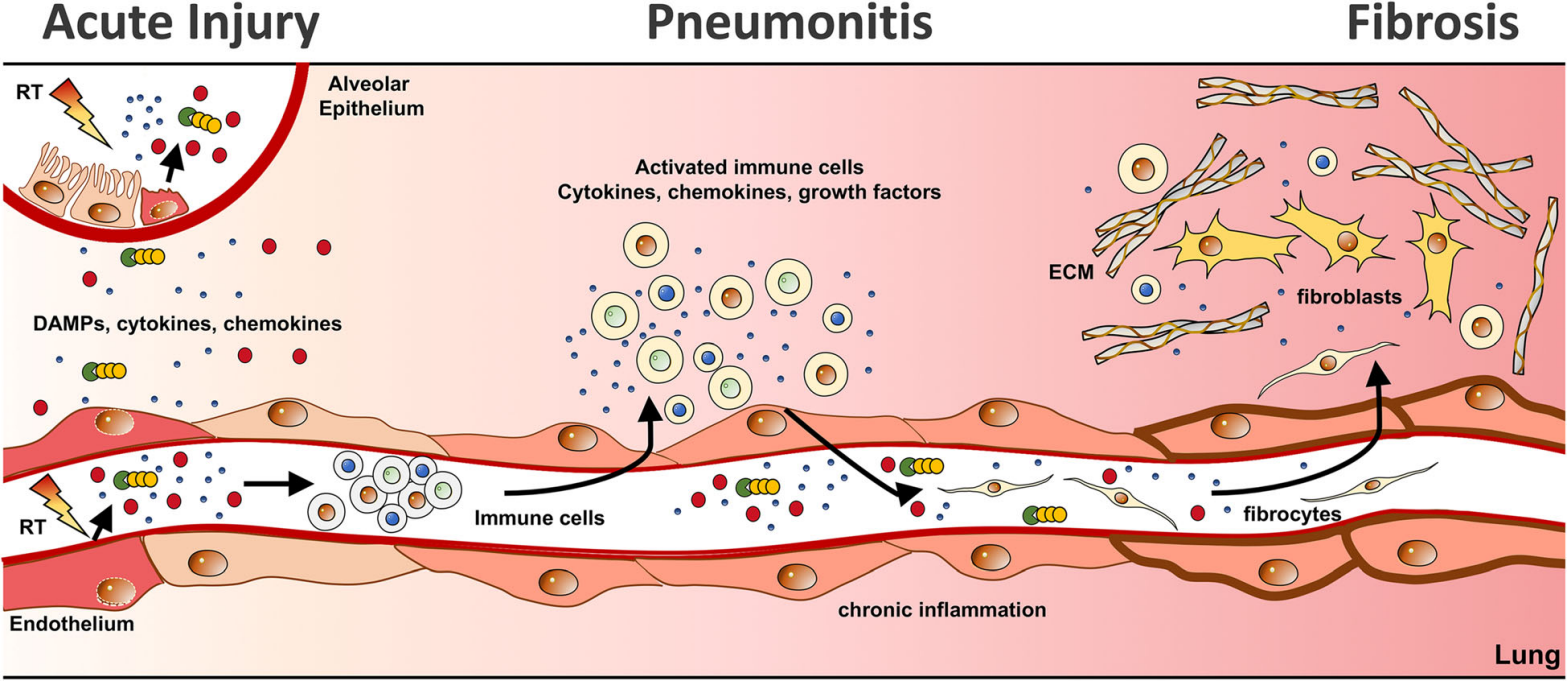
Schematic illustration showing the progression of radiation-induced pneumopathy. Radiotherapy (RT) of the thoracic region can induce damage and death in epithelial and endothelial lung cells. Subsequent release of DAMPs, cytokines and chemokines leads to the recruitment of diverse immune cells into the lung tissue. An overwhelming cascade of pro-inflammatory cytokines secreted by activated immune cells can result in radiation-induced pneumonitis. During the progression of radiation-induced pneumopathy chronic inflammation, tissue hypoxia and growth factor release result in further microenvironmental changes in the lung tissue. Recruitment of fibrocytes and secretion of pro-fibrotic mediators trigger differentiation of fibroblasts from various sources including mesenchymal stem cells [250] and excessive deposition of extracellular matrix molecules (ECM) resulting in lung fibrosis

Depending on the radiation dose and the irradiated volume damage to resident cells with induction of senescence or cell death [12, 19, 20] initiates an acute damage response with release of danger signals, that are recognized by specialized receptors and culminate in the creation of a multifaceted pro-inflammatory and pro-angiogenic microenvironment [21] (for more details see Fig. 1 and below). Cytokines and chemokines subsequently trigger waves of infiltrating immune cells and can result in a pronounced interstitial pneumonitis in the irradiated as well as in non-irradiated areas of the lung [3, 4]. Activation of conserved damage response programs by therapy-induced cell death are also known to contribute to regeneration or even fibrosis development in normal tissues but can also promote repopulation in tumor tissues. Delayed senescence or mitotic cell death of resident cells at later stages may therefore shape further progressive changes in the lung environment that are reminiscent of an exaggerated wound healing response such as tissue hypoxia and chronic inflammation, accumulation of growth factors, proteases and other pro-fibrotic mediators, excessive deposition of extracellular matrix molecules and may culminate in the development of lung fibrosis months after RT [3, 8–10] (see Fig. 1).

The current view on the molecular and cellular pathways of the DNA damage response (DDR) that drive activation of the immune system and the mechanisms how DNA damage drives chronic inflammation and vice-versa has been summarized elsewhere [22, 23]. Moreover, senescence and the senescence-associated secretory pattern (SASP) recently emerged as further important drivers of tissue inflammation and repair. In this context, animal models of DNA damage induced lung disease such as exposure to IR [24, 25], BLM-treatment [26, 27] or telomere dysfunction [28] revealed that induction of senescence is an important process in alveolar epithelial cells (AEC) as well as myofibroblasts exposed to DNA damage. These studies revealed that accumulation of senescent AECs or myofibroblasts result in apoptosis resistance and secretion of SASP-factors and this was associated with fibrosis development. Even more important, targeting the senescence-driving NADPH oxidase (Nox4)/NFE2-related factor 2 (Nrf2) axis [26] or clearing the apoptosis-resistant or senescent cells by inhibition of anti-apoptotic BCL-2/X_L_ [25] or so-called “senolytic” agents [27] attenuated the senescent phenotype as well as fibrosis development in the murine models of thoracic irradiation [25] and BLM treatment [27], respectively. But, we are only beginning to understand the complex effects of certain aspects of senescence in the context of DNA damage-induced injury and will not go further into detail here since the link between DNA damage, senescence, inflammation, and cancer has been nicely summarized elsewhere [29–32].

Preclinical investigations in rodent models revealed that depending on the radiation dose and the irradiated volume a single high dose irradiation to the thoracic region can induce pneumonitis, fibrosis or both in rats and mice [12, 33–35]. Importantly the tissue response to radiation well depends on the genetic background: For example, of the various murine strains only the C57 strains such as C57BL/6 mice and C57L mice develop both, prominent pneumonitis and lung fibrosis, upon a single high dose irradiation to the whole thorax (WTI) or the right hemithorax (HTI), whereas C3H, BALB/c, A/J and CBA mice are prone to develop pneumonitis but are fibrosis-resistant [36–39].

The availability of various knockout models on the C57BL/6 background and the similarities in the time course and the observed symptoms between this murine model and the human disease makes C57BL/6 mice the most frequently used model to investigate radiation-induced adverse late effects in the lung to date [40]. Interestingly, mortality upon WTI seems to be sex-dependent, at least in mice as increased median survival times upon high dose WTI have been observed in male mice of several studies [41, 42]. This has recently been summarized by Dabjan et colleagues in a review about the use and the limitations of studies about radiation induced pneumopathy in various inbred mouse strains [40]. The authors also pointed out that the vast majority of all murine studies use high dose WTI or total body irradiation (TBI); such a high dose radiation exposure of the whole lung is reminiscent for example of myeloablative conditioning regimens in patients prior to hematopoietic stem cell transplantation and severe pneumonitis is considered as major risk factor for dose-limiting toxicity in humans receiving such a systemic irradiation [8, 40]. In contrast, hemithoracic irradiation (HTI) may constitute a more appropriate model for a localized regional pulmonary irradiation that is commonly used for therapeutic irradiation of cancer patients; sensitive patients can develop symptomatic or fatal pneumonitis and/or pulmonary fibrosis depending on the dose, fractionation scheme and the irradiated volume [40, 43–45]. But C57BL/6 mice develop radiation-induced pneumonitis at 3–12 weeks and fibrosis at 24–30 week post-irradiation upon both, HTI or WTI so that despite some advantages of the HTI model, both models are suited to gain insight into the mechanisms underlying radiation-adverse late effects in the lung. Of course, it is highly desirable that the above-mentioned models of single high dose irradiation will also be complemented by investigations with standard fractionation, as well as hypo- and hyper-fractionated irradiation schedules that are commonly used in cancer treatment.

### Modeling adverse late effects in the lung by treatment with the radiomimetic drug BLM

Due to limited availability of equipment for IR and other restrictions, the murine model of BLM-induced pneumopathy has also been used to investigate DNA-damage induced adverse late effects in the lung as well as for studying the mechanisms underlying the pathogenesis of pulmonary fibrosis per se [46–48]. Herein the BLM model has the advantage that the symptoms of pneumonitis and pulmonary fibrosis develop much faster, namely within 5 weeks in C57BL/6 mice, depending on the route of administration (Table 1) [46, 48, 49].

**Table 1:** Summary highlighting the differences between the murine models of DNA-damage induced pneumopathy of thoracic irradiation and Bleomycin (BLM) treatment

Thoracic Irradiation	Bleomycin	
-Model of chronic lung damage -Single hemi thorax or whole thorax irradiation with 12–20 Gy -Damage to the whole tissue (endothelial, epithelial, immune cells) -Induction of stress or cell death initiates an acute damage response -Interstitial pneumonitis at week 3–12 -Senescence or mitotic cell death - > Chronic inflammation -Sub-pleural and bronchiolocentric fibrosis at week 24–30	intratracheal/oral	-Model of acute lung damage -Single BLM instillation. 1–5 U/kg -Direct and primary damage to the alveolar epithelium -Rapid pro-inflammatory immune response -Excessive fibroblast proliferation and extracellular matrix deposition -Fibrotic lesions are concentrated in the bronchiolocentric region -Fibrosis development as of day 14 -Peribronchial interstitial fibrosis is reversible and self-limiting after 28 days
	intraperitoneal/intravenous	-Model of chronic lung damage -Repetitive BLM injections. 15-50 U/kg -Primary damage to endothelial cells of capillaries and blood vessels and perivascular structures of the sub-pleural parenchyma -Secondary damage to the alveolar epithelium -Acute inflammatory response within the first week -Fibrosis development as of day 9 -Prominent sub-pleural fibrosis at day 28–35

The antibiotic peptide BLM, isolated from *Streptomyces verticillus*, has already been described in the 1960s as a drug with anticancer activities and was thus used for cancer treatment [50]. Since the early 1970s BLM was used in the clinics to treat cancer patients in combination with radiotherapy [51]. However, during this time researchers already observed, that BLM alone or in combination with radiotherapy has strong toxic effects on the normal tissue, particularly in the skin and the lung [49, 52, 53]. The susceptibility of the lung towards BLM is attributed to reduced BLM hydrolase levels in this organ [54]. The BLM hydrolase is an intracellular cysteine protease and usually metabolizes BLM leading to its inactivation [55]. For that reason, BLM-induced pulmonary damage became a common model to study pneumonitis and pulmonary fibrosis by treating animals either by subcutaneous [56] or intravenous injections [52] or intratracheal instillation of BLM [57], respectively.

Similar to IR, BLM exerts its cytotoxic effects mainly by inducing single and double strand breaks in the DNA [58] and by inducing cell death [59, 60]. BLM has two structural sites, one binding to the DNA and one binding to oxygen and iron thereby forming an activated complex [61]. Initially it was thought that the cytotoxic effect is only dependent on oxygen and the formation of DNA-damaging hydroxyl radicals [62, 63], but the current opinion is that BLM also exerts its effects by direct cleavage of DNA strands through abstraction of H^+^ protons [64–67]. Further toxic effects of BLM involve degradation of unsaturated fatty acids in the cell membrane [68] and enhanced lipid peroxidation [69], at least in vitro. Interestingly, it has also been proposed that BLM may bind to receptors on cells thereby inducing Toll-like Receptor (TLR) signaling or BLM uptake [70–72] and subsequent immune responses [73–76].

Importantly, preclinical investigations in mice and rats implicate that the tissue response to BLM and particularly the immune-related drug effects may depend from its administration route: The common single intratracheal application mostly induces a rapid and prominent pneumonitis with subsequent reversible fibrosis peaking at day 14 [38, 47, 77, 78]. In contrast, repeated intraperitoneal injections trigger an acute inflammatory response within the first week that is followed by a switch to fibrosis development at day 9 and massive fibrosis at days 28–35 [47, 77, 79].

Based on these findings, it had been speculated in the 80s that intratracheal and systemic administration of BLM might elicit distinct immune responses due to the fact that the pulmonary environment might perceive an inhaled foreign substance differently compared to a substance entering the lung via the circulation [80]. The mucosal barrier of the lung is permanently exposed to several non-harmful and harmful inhaled compounds. Furthermore, the respiratory tract is inhabited by specific respiratory microbiota that are thought to provide resistance against respiratory pathogens and might also participate in building and maintaining lung homeostasis and immunity [81, 82]. The pulmonary immune system thus needs to fulfill a balance between tolerance and inflammation. Inhaled BLM might alter this balance more prominently thereby inducing a more severe inflammation and eliciting more pronounced tissue damage.

Nowadays we know that the route of BLM administration results in important differences in the degree of inflammation as well as in the distribution of the resulting fibrotic lesions. It has been demonstrated that a single intratracheal instillation of BLM results in a direct and primary damage to the alveolar epithelium that is followed by a rapid pro-inflammatory immune response leading to additional damage to AECs. Excessive fibroblast proliferation and extracellular matrix deposition finally result in the development of reversible fibrosis already at 2 weeks after BLM treatment [83]. Therefore, intratracheal BLM administration is considered as a model of acute lung damage. Because of the massive primary damage to AECs the fibrotic lesions upon intratracheal BLM administration are concentrated in the bronchiolocentric region, resulting in peribronchial interstitial fibrosis [38, 46, 48, 77]. But a novel model of repetitive intratracheal instillation has meanwhile been established that should reflect more accurately the chronic disease state of pulmonary fibrosis in patients [84, 85].

In contrast, repetitive intravenous or intraperitoneal BLM-injections are thought to represent chronic models of DNA damage-induced lung injury [79]. It has been proposed that in these models the primary damage to resident lung cells mainly concerns the endothelial cells of capillaries and blood vessels as well as perivascular structures of the sub-pleural parenchyma and is followed by secondary damage to the AEC, tissue inflammation and collagen deposition. Thus, a sub-pleural fibrosis is usually observed in these settings [46, 48].

### Tissue damage, danger signals and immune activation

The ability of X-rays to modulate immune responses and to stimulate lymphocyte responses has already been revealed about 100 years ago [86, 87]. But only the discovery that mammalians detect microbial infection (presence of dangerous, infectious non-self) [88] as well as injury (presence of dangerous self) [89] by so-called pattern recognition receptors allowed us to understand why and how exposure to ionizing radiation and other physical stressors activate conserved host defense signaling pathways including immune activation and may thereby act as immune adjuvants. In 1994 Polly Matzinger and colleagues introduced the concept of danger-induced immune activation describing the ability of the immune system to respond to alarm signals released from damaged tissues [89, 90]. In analogy to the so-called *pathogen-associated molecular patterns* (PAMPs) these host alarmins have been named *damage-associated molecular patterns* (DAMPs) though some categories of DAMPs such as nucleic acids (DNA, RNA) even encompass foreign as well as host alarmins [91].

Nowadays it is widely accepted that tissues control the initiation of a damage response by sensing danger signals released by stressed, damaged, or dying cells. Ubiquitous DAMPs include for example extracellular ATP, extracellular DNA, *high mobility group box chromosomal protein B1* (HMGB1), *heat shock protein 70* (HSP70), uric acid, and fragmented extracellular matrix molecules such as low molecular weight hyaluronan (HA). These molecules are normally hidden intracellularly or masked for example by chaperones, aggregate formation, or membrane insertion. But upon release, unfolding or activation these DAMPs signal tissue injury to the host and initiate processes that allow for repair and reconstitution of tissue homeostasis [91–96].

Exogenous and endogenous danger signals are perceived by innate pattern recognition receptors (PRR) such as membrane-bound *TOLL-like receptors* (TLR) as well as cytoplasmic *nucleotide binding and oligomerization domain* (NOD)-like receptors (NLR), *receptor for advanced glycation end products* (RAGE), *C-type lectin receptors* (CLR) or *retinoic acid-inducible gene-1 (RIG-1)-like receptors* (RLR) [97–102]. Inflammasomes constitute another facet of immune activation upon danger [103]. These cytosolic protein complexes are formed upon exposure to PAMPs or DAMPs and enable activation of the inflammatory protease caspase-1. Caspase 1-activation subsequently catalyzes proteolytic cleavage and release of the pro-inflammatory cytokines interleukin (IL)-1β and IL-18, a specialized form of cell death named pyroptosis, or both [104–106]. Instead, the prototypical danger molecule ATP signals via P2 nucleotide receptors presumably P2X_7_ [107, 108]. DAMP signaling of these diverse receptors has been covered in detail in general reviews about immunological responses in inflammation, cancer and tissue repair and will not be described here [94, 102, 103, 109–114]. We will also not include investigations about immune activation by cyclic GMP-AMP synthase (cGAS) and STING-dependent sensing of cytosolic DNA that turned out to be important for radiation-induced immune enhancement in tumors and tumor regression [115, 116] as the role of the respective signaling molecules has not yet been investigated with respect to the damage response in normal tissues.

The initial concept that the release of danger signals from injured tissues dictates immune activation was later expanded to the view that tissue-derived signals also control the effector class of the resulting immune response in order to maintain or reconstitute the physiological microenvironment and preserve tissue function [117]. The release of danger signals upon injury will be influenced by the specific and preferred local communication of the respective tissue with the immune system, e.g. the activation of resident or recruited antigen-presenting cells or immigrating populations of innate lymphocytes, as well as the locally available DAMPs, DAMP receptors and responsive factors. For example, the immunosuppressive cytokine *transforming growth factor β1* (TGFβ1) is constitutively expressed by many lung cell types including lung epithelial cells and these cells also express the integrins required to convert latent TGFβ1 into its active form to maintain immune homeostasis under homeostatic conditions [118].

We assume that the tissue state at the time of injury will impact the tissue response to injury as well. Otherwise, the extent and type of damage will certainly influence the damage response; under certain conditions the damaging agent may even affect the damaged tissue in a way that the resident cells become unable to properly control innate and adaptive immune responses or activate repair-promoting processes as well as the involved regulatory signaling network. Such a disturbed signaling network may lead to insufficient or exaggerated inflammation, immune suppression or impaired inflammatory resolution, or even pathologic remodeling. In this context, tissue responses to persistent damage frequently lead to deregulated wound healing processes that finally result in the development of tissue fibrosis. Since chronic inflammation is a key component of such wound healing processes it is thought to participate in the pathogenesis of tissue/organ fibrosis [111, 119] and even cancer [120].

Various damage and injury models have meanwhile been described that involve DAMP-induced or inflammasome-induced sterile inflammation mediated for example by TLR signaling e.g. renal ischemia/reperfusion injury [121], allograft reperfusion injury after transplantation [122] trauma [123, 124], gout [125] and silica/asbestos induced injury [126] and these acute inflammatory pathways are also important to the development of organ fibrosis [103, 119]. Importantly, danger signals from irradiated tissues can also activate downstream immune effector pathways to support cancer cure, or modulate acute and late adverse radiation effects (for a detailed review see [1, 127]). Although several studies investigated the roles of additional PRR in BLM-induced fibrosis, e.g. RAGE [128], so far only the role of single TLR has been investigated with respect to radiation-induced pneumopathy (see below). Therefore, here we focus on TLR signaling and associated processes.

### TOLL-like receptor (TLR) signaling

TLR are conserved receptors for the recognition of conserved exogenous infectious microbial structures (PAMPs) and endogenous DAMPs that are released by damaged cells and injured tissues and are able to activate antigen-presenting cells [91]. Activation of TLR induces the production of pro-inflammatory cytokines (e.g. TNFα, IL-1β, IL-18, IL-6, and IL-12), chemokines and Type I interferons as well as the maturation of antigen-presenting cells that subsequently prime naive T cells and elicit specific T−/B-cell immune responses. Thus, TLR signaling is a crucial step in host defense and immunity against microbial and non-infectious injury in mammalian cells [129]. Furthermore, based on their function in the tissue response to damage TLR can also promote tissue repair and even participate in the development of tissue fibrosis in various models, but with profound differences between the damaged tissues/organs. Interestingly, the effect of TLRs on the pathogenesis of tissue fibrosis is most prominent in tissues with pronounced and constant exposure to bacterial TLR ligands, such as the liver and gut and - to a lower extent - the lung [119]. Most mammalian species have 10 to 13 types of TLRs that recognize specific foreign (non-self) or endogenous ligands (dangerous self) and induce various inflammatory cascades [130]. Of the 10 TLRs found in humans six are located at the extracellular membrane (TLR1, TLR2, TLR4, TLR5, TLR6, and TLR10) and four are located intracellularly at the endosomal membrane (TLR3, TLR7, TLR8, and TLR9) to allow detection of DAMPs at their preferred trafficking paths. The diverse TLR differ in the extracellular ligand-binding domain to allow detection of the various ligands, as well as the use of adapter proteins and associated kinases for the activation of transcription factors [131, 132]. Consequently, loss of specific TLR will disturb specific aspects of infection-induced or damage-induced activation of innate and adaptive immune responses whereas artificial activation of TLR signaling can help the tissue in the protection against damage and the acquisition of tolerance to infection or injury (see also final remarks).

**Fig. 2:**
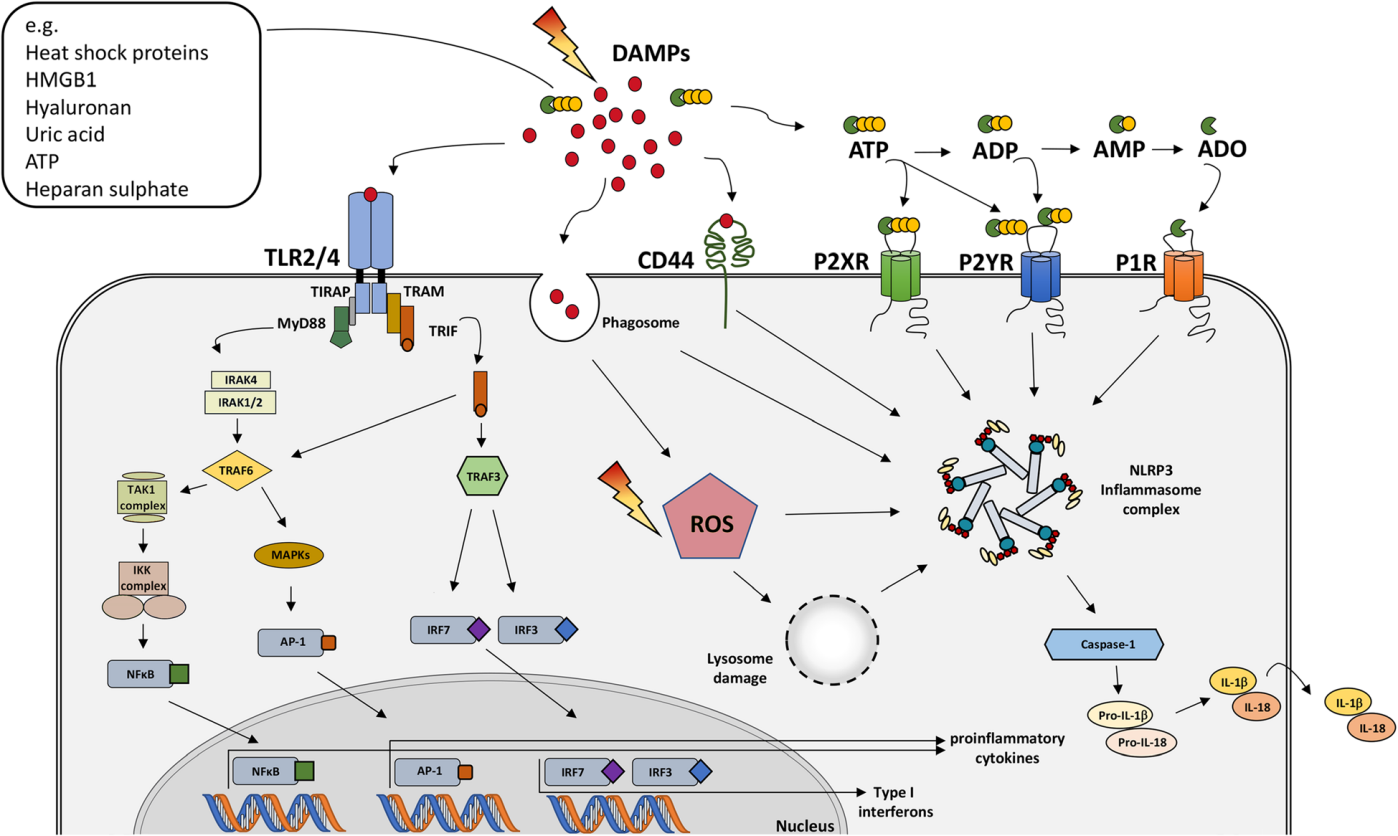
Schematic illustration of DAMP/ TLR/ inflammasome interactions. Damage-induced release of damage associated molecular patterns (DAMPs) into the extracellular region can result in ligand binding to specific receptors or uptake in diverse target cells. TOLL-like receptor (TLR)-binding and recruitment of the adaptor protein MyD88 leads to subsequent activation of TNF receptor associated factor (TRAF) 6 in the cytosol. Activation of the IKK kinase complex or *mitogen-activated protein kinase* (MAPKs) result in the translocation of the transcription factors n*uclear factor kappa B* (NF-κB) and a*ctivator protein 1* (AP-1) into the nucleus respectively. In the nucleus they induce the expression of pro-inflammatory cytokines like pro-IL-1β/ IL-18. TLR4 internalization will induce a switch from MyD88 to *TIR-domain-containing adapter-inducing interferon-β* (TRIF) signaling [135, 288]. The recruitment of TRIF results in signaling cascades similar to the ones described for the MyD88 pathway. On the one hand, TRIF can either interact with TRAF6 resulting in NFκB-dependent or AP-1 dependent production of inflammatory cytokines. In addition, TRIF can also interact with TRAF3 leading to the induction of the transcription factors *Interferon regulatory factor* (IRF)3/7. Translocation of IRF3/7 into the nucleus then triggers the production of type 1 interferons [130–134]. Furthermore, reactive oxygen species (ROS) and DAMPs can induce the activation of the NLR family, pyrin domain-containing 3 (NLRP3) inflammasome complex and subsequent caspase-1 activation, capable of cleaving pro-IL-1β and pro-IL-18 into mature and secreted IL-1β and IL-18. Nucleotides like ATP, ADP, AMP and the nucleoside adenosine can bind to its purino-receptors like P2XR, P2YR and P1R, respectively, thereby triggering the activation of the NLRP3 inflammasome complex. Additionally, intracellular DAMP (e.g. uric acid) uptake into phagosomes, lysosomal damage or specific binding of e.g. hyaluronan to the CD44 receptor can further activate the NLRP3 inflammasome complex, promoting inflammation

TLR are membrane-integrated glycoproteins with characteristic ligand-binding extracellular moieties and a common cytoplasmic TLR/IL-1 receptor (TIR) signaling domain [130]. Ligand-binding leads to homo- or hetero-dimerization and subsequent activation of two major downstream pathways that differ in the respective adapter proteins: The first pathway is mediated by *myeloid differentiation primary response factor 88* (MyD88) without and with additional requirement of *TIR-domain containing adapter protein* (TIRAP), whereas the second pathway involves the adapter protein *TIR-domain-containing adapter-inducing interferon β* (TRIF) without and with additional requirement of *TRIF-related adaptor molecule* (TRAM) [133] (see Fig. 2). TIRAP is also important to MyD88 recruitment by TLR1/2 und TLR2/6 dimers [131]. All known mammalian TLR except TLR3 use MyD88-dependent pathways, whereas TLR3 exclusively activates TRIF-dependent pathways, and TLR4 is unique in this regard as it can use all four adapter proteins (TRAM, TRIF, TIRAP, MyD88) to signal infection and injury via both pathways [133].

The main effector pathways of TLR signaling include activation of mitogen-activated protein kinase (MAPK) and nuclear factor of kappa light polypeptide gene enhancer in B cells (NF-κB) signal transduction pathways as well as interferon regulatory factors IRF3 and IRF7; these regulate cell differentiation, proliferation, and survival as well as production of type I interferons [130–134]. Interestingly, the choice of the signaling paths depends on the subcellular localization of the respective TLRs, at least for MyD88-dependent pathways [135].

### Importance of damage signaling and resulting immune changes for the pathogenesis of DNA damage-induced pneumopathy

Infiltration of immune cells is a common finding in irradiated tissues. This is not surprising since activation of the innate and adaptive immune systems is part of the tissue response to damage or injury as outlined above. Thereby danger signals released by damaged tissues or bystander cells outside the radiation field participate in mediating either acute/chronic disease, recovery from radiation-induced damage, or even tolerance to subsequent injury [136]. However it is highly likely that each tissue/organ may use unique sets of signaling molecules and effector cells to orchestrate the local response to injury [117]. So far, the role of damage-induced immune activation in balancing tissue regeneration versus chronic lung disease is not well understood. This situation is further complicated by the fact that the cells executing innate immune responses comprise both, professional immune cells as well as nonimmune cells [124], e.g. alveolar epithelial type II (AECII) cells in the lung [137–140]. Since epithelial cells constitute a major part of the cells in a given organ, their relative contribution to injury may be substantial [124].

Though it is widely accepted that irradiated cells and tissues release danger signals to the immune system, the respective danger signals and TLR or other pattern recognition receptors involved in the radiation response of specific tissues including the lung remain elusive [141]. The same holds true for the contribution of specific immune cell types [142]: So far our conclusions are based on the use of a very limited set of assays and surface/response markers to unravel pathogenic mechanisms that are controlled by a complex communication network of cross-regulatory signaling pathways between distinct cell types including various versatile cells from the innate and adaptive immune systems. These cells can shape their behavior according to microenvironmental changes and adopt both, disease promoting and protective functions by a switch in their phenotype [91]. This makes their interpretation and sound conclusions difficult.

We suppose that radiation-induced and BLM-induced pneumonitis and pulmonary fibrosis constitute two further facets of injury-induced immunity. DAMPs not only signal danger but also participate in the regulation of tissue repair after sterile or infection-associated inflammation [111]. Thus, DAMPs released from damaged lung tissue will play complex roles in the damage response to WTI or BLM since they may balance excessive pneumonitis versus inflammatory resolution as well as tissue repair versus pulmonary fibrosis, respectively. This might explain why the extent and type of damage as well as the type of primary damaged cell impact the tissue response to radiation-induced or BLM-induced injury and thus treatment outcome. It will therefore be important to identify the DAMPs that specifically signal excessive radiation-induced or BLM-induced injury to the host, the receptors that perceive the released DAMPs, the preferred signaling pathways that communicate the damage signals to the innate immune system as well as the resulting adaptive response if we aim to modulate disease outcome by interfering with therapy-induced immune deviation.

Mutations in signaling pathways that are responsible for the communication of radiation-induced or BLM-induced lung damage and repair will impact the outcome of sterile inflammation followed by inflammatory resolution and regeneration versus immune deviation and pulmonary fibrosis. Consequently, the use of mice deficient in suspected signaling molecules is an important approach to test the contribution of specific signaling pathways. Here, we performed a critical review of the findings obtained in studies with C57BL/6 knockout mice with specific defects in danger signaling/immune regulation and in immune-deficient inbred murine strains to gain a better understanding of the contribution of danger signaling and resulting immune changes to the development of radiation-induced and BLM-induced pneumonitis and pulmonary fibrosis, and to reveal what we can learn from this for future studies. These data mostly come from studies that evaluated the effects of WTI or intratracheal BLM administration and include investigations in C57BL/6 knockout mice with deficiency in TLR2, TLR4 or MyD88, CD73 or interleukin-6 (IL-6) and IL-4 or with alterations in B−/T-cells and are summarized in Table 2.

**Table 2:** Studies analyzing the effects of specific defects in danger signaling/immune regulation after whole thorax irradiation or Bleomycin treatment

Strain	Whole thorax irradiation	Ref.	Bleomycin	Ref.
TLR2^−/−^	- 18Gy, endpoint 26wks - pneumonitis and fibrosis development like WT	Paun	- intratracheal, 2 U/kg, endpoint 21d - reduced BALF cells - enhanced BALF IL-17 level - reduced BALF TGF-b and IL-27 - reduced fibrosis development	Kim
			- intratracheal, 3 U/kg, endpoint 28d - reduced M1/M2, Treg and pDC - reduced HMGB1 and TGF-β - reduced apoptosis of pulmonary cells - reduced fibrosis developemnt	Yang
TLR4^−/−^	- 18Gy, endpoint 26wks - pneumonitis and fibrosis development like WT	Paun	- intratracheal, 3 U/kg, endpoint 28d - enhanced inflammatory cells - enhanced immunosuppressive cytokines - attenuation of pro-infl. p38 signaling - activation of immunosuppressive ERK signaling - enhanced fibrosis developemnt	Yang
TLR2/4^−/−^	- 18Gy, endpoint 26wks - same infiltration compared to WT - same apoptosis induction like WT - enhanced fibrosis development	Paun	- intratracheal, 5 U/kg, endpoint 21d - reduced survival - reduced BAL cells and PMN - enhanced apoptotic cells - enhanced inflammation	Jiang
MyD88^−/−^	- 14Gy, endpoint 27wks - enhanced TH2 cells - enhanced fibrosis development	Brickey	- intratracheal, 5 U/kg, endpoint 21d - reduced BAL cells and PMN - enhanced apoptotic cells - enhanced inflammation	Jiang
			- nasal, 15 U/kg, endpoint 21 d - reduced lymphocytes, neutrophils, macrophages - reduced pro-infl. cytokines - reduced TGF-β, TIMP-1 and MMP2/9 - reduced inflammation and fibrosis	Gasse
CD73^−/−^	- 15Gy, endpoint 25-30wks - reduced adenosine in BALF - reduced early apoptosis - reduced pro-fibrotic factors - reduced fibrosis development	Wirsdorfer	- intratracheal, 3.5 U/kg, endpoint 14d - reduced adenosine in BALF - enhanced lymphocytes, neutrophils, macrophages - enhanced pro-infl. and pro-fibr. factors - enhanced collagen and fibrosis	Volmer
IL-6^−/−^	- 10Gy, endpoint 4wks - stable CD44 and Bak level compared to WT (enhanced) - same inflammatory foci induction compared to WT	Sakai	- intratracheal, 1 U/kg, endpoint 21d - reduced acute neutrophil infiltration (d2) - reduced BAL cell counts (d2, d21) - reduced collagen and fibrosis	Saito
	- C-lon 10Gy, endpoint 24wks - same macrophage infiltration at week 12, 24 - reduced fibrosis development	Saito-Fujita		
IL-4^−/−^	- 12.5 Gy, endpoint 26wks - reduced M2 macrophages - fibrosis development like WT	Groves	- intratracheal, 1 U/kg (2.5 U/kg), endpoint 28d - 100% mortality at d12, 2.5 U/kg treated mice - reduced eosinophils, neutrophils, macrophages at d7 - reduced lymphocytes at d14 - reduced collagen, fibronectin, fibrosis at d28	Huaux
			- intratracheal, 2.2 U/kg, endpoint 14d - same BAL cell level compared to WT - enhanced z score values for hydroxyproline, fibrosis fraction	Izbicki
			SCID mice - intratracheal, 3 U/kg, endpoint 21d - fibrosis development like WT	Lake-Bullock
RAG2^−/−^, athymic NudeSCID	RAG2^−/−^ mice	Cappuccini	athymic Nude mice -intratracheal, 3 U/kg, endpoint 14d - reduced lung collagen synthesis compared to WT	Schrier
	- 15Gy, endpoint 24wks - prominent fibrosis already in week 24 p.l.		athymic Nude mice "low" dose intraperitoneal 8 times ﻿- 20 U/kg 2 times/week, endpoint 8wks - mild alveolitis and fibrosis athymic Nude mice "medium" dose intraperitoneal 8 times - 50 U/kg, 4 times/week, endpoint 4wks- prominent alveolitis and fibrosis	Szapiel

*Gy* Gray, *p.I* post irradiation, *U* units, *BALF* bronchoalveolar lavage fluid, *pDC* plasmacytoid dendritic cells, *PMN* polymorph nuclear leukocytes, *WT* wildtype

### Role of TLR signaling in the damage response of the lung tissue

As described above TLR are germ line-encoded receptors for the detection of conserved microbial structures and host alarmins released from damaged tissues*.* TLR activation signals immune activation in professional antigen-presenting innate immune cells but also non-immune cells which subsequently promotes/shapes the activation of T−/B-cell responses [119]. The main functions of TLR in the respiratory tract during acute and chronic lung inflammation have been summarized in detail elsewhere [143]. TLR2 and TLR4 seem to be particularly important for mediating acute and chronic effects of non-infectious tissue damage in the lung through detection of endogenous DAMPs and subsequent activation of inflammatory immune responses. Thus, several publications revealed a contribution of TLR2 or TLR4 in non-infectious acute lung injury (ALI), acute respiratory distress syndrome (ARDS), chronic obstructive pulmonary disease (COPD), cystic fibrosis (CF) and asthma [144–147]. Based on their function in promoting tissue repair upon injury TLR were also shown to play a role in lung fibrosis in various damage models [119].

Tissue resident cells of the innate immune system, e.g. alveolar macrophages and dendritic cells and potentially innate lymphoid cells, as well as their recruited relatives, e.g. infiltrating monocytes, neutrophils, NK cells or γδ T cells, express TLR and provide the first line of defense upon tissue injury but are also critical in regulating recovery from injury, tissue repair and fibrosis [119, 142]. For example neutrophils, monocytes and macrophages respond to injury-induced changes in their microenvironment by phenotypical and functional changes to exert their multiple functions during tissue repair such as phagocytosis of cell debris, production or degradation and removal of ECM components, and secretion of growth factors and mediators that modulate inflammation and repair. But maladaptive macrophage responses can result in deregulated production of matrix-degrading enzymes, growth factors, pro-inflammatory, anti-inflammatory or profibrotic mediators and disturbance of the delicate communication network between macrophages and epithelial cells, endothelial cells, fibroblasts, and tissue-resident progenitor cells thereby promoting persistent tissue damage and the development of fibrosis (for a detailed review see [148, 149]). This makes macrophages an attractive target for fibrotic lung disease including radiation-induced pulmonary fibrosis [13, 150]. With respect to TLR signaling it was shown that *IL-1R-associated kinase-M* (IRAK-M), a MyD88-dependent inhibitor of TLR signaling, suppressed lung inflammation, but paradoxically promoted the development of pulmonary fibrosis in mice presumably by promoting an alternatively activated profibrotic phenotype of macrophages [151]. We therefore believe that balancing the pathologic phenotype of macrophages may be a better approach to prevent or treat fibrotic disease caused by chronic injury.

But resident epithelial cells, endothelial cells as well as fibroblasts and other non-myeloid resident cells also express TLR and impact injury-induced inflammatory and repair processes as described in detail elsewhere [119]. For example, pulmonary fibroblasts respond to exogenous DAMPS such as LPS or unmethylated CpG DNA via TLR4 and TLR9 respectively [152–157]. Of note, under physiological conditions pulmonary epithelial cells are in permanent contact to commensal bacteria; but pulmonary epithelial cells are also exposed to inhaled irritating or toxic compounds or pathogenic bacteria and thus have to balance tolerance to commensal bacteria versus protection against pathogens and toxic compounds. There is increasing evidence that commensal bacteria present in the respiratory tract induce tissue-protective factors in epithelial cells e.g. via TLR4/MyD88, and that loss of TLR4/MyD88 signaling may reduce injury-induced inflammation but impair proper repair [158]. This might explain why the respiratory microbiome is critical for epithelial homeostasis and impacts the development of lung fibrosis as shown in patients with idiopathic pulmonary fibrosis [81, 119, 159, 160].

Taken together TLR seem to play a critical role in the complex signaling network between resident cells and recruited immune cells in regulating the tissue response to injury, tissue regeneration and chronic fibrotic disease in the lung. However, the contribution of specific TLR to DNA damage-induced pulmonary fibrosis and the contributing cell types are less clear.

### Role of TLR2 or TLR4 signaling in DNA damage-induced pneumopathy

So far, a role of TLR2 and TLR4 has been described for DNA-damage induced adverse late effects in the lung with partially controversial findings in the WTI and BLM models. Paun and colleagues analyzed the effects of 18Gy (Gray) WTI on the development of pneumonitis or fibrosis in mice deficient for TLR2 (TLR2^−/−^), TLR4 (TLR4^−/−^), and TLR2/4 double knockout mice (TLR2^−/−^/TLR4^−/−^) [161]. The observation that the lung phenotype of TLR2^−/−^ or TLR4^−/−^ mice did not differ from C57BL/6 wildtype (WT) mice revealed that the single knockout mice (TLR2^−/−^ or TLR4^−/−^) have similar sensitivity to WTI-induced alveolitis and fibrosis as WT mice. However, combined loss of TLR2 and TLR4 in TLR2^−/−^/TLR4^−/−^ enhanced WTI-induced fibrosis compared to WT mice [161]. This suggested that combined signaling via both receptors is required to provide TLR-mediated tissue protection, or alternatively that TLR2 and TLR4 may have a redundant function herein.

In line with these findings, TLR2^−/−^/TLR4^−/−^ were also more susceptible to pulmonary fibrosis induced by acute intratracheal BLM administration [158]. Increased fibrosis was linked to reduced production and reduced transepithelial migration of neutrophils. Furthermore, apoptosis in epithelial cells was increased resulting in chronic inflammation and fibrosis. Interestingly, the authors could demonstrate that TLR2 and TLR4 were necessary to promote tissue-protective effects of the extracellular matrix glycosaminoglycan hyaluronan (HA) by inducing optimal chemokine production and stimulating NF-κB-mediated protection of epithelial cells from BLM-induced apoptosis [158].

In contrast to the WTI model, single deficiency of TLR4 in TLR4^−/−^ mice also led to a massive inflammation and more pronounced pulmonary fibrosis upon intratracheal BLM application and this was linked to more pronounced lung dysfunction and increased death of the TLR4^−/−^ mice compared to WT mice [162]. Accordingly, pharmacologic activation of TLR4 with an activating anti-TLR4 antibody reduced inflammation and reversed the pathogenic effects of intratracheal BLM administration [162]. These observations highlight a critical role of TLR4 signaling for the resolution of acute inflammation and protection against the adverse effects of BLM in the lung.

Surprisingly, single deficiency of TLR2 in TLR2^−/−^ mice reduced lung inflammation and damage upon intratracheal BLM administration leading to attenuated pulmonary fibrosis [75, 163]. Thus, in contrast to the protective action of TLR4, TLR2 has disease-promoting effects in the acute model of BLM-induced pulmonary fibrosis. We assume that this may be linked to the suggested action of BLM as an agonist of TLR2 [76]. Accordingly, a specific agonist of TLR2 induced potent T_H_1 responses and severe lung injury in the acute model supporting the disease-promoting effects of TLR2 in this model [75]. Yang and co-workers showed that the protective effects of TLR2-deficiency were accompanied by lower SMAD3/STAT3 activity and reduced levels of T_reg_, M2 macrophages and TGF- β during disease pathogenesis. The authors thus concluded that DAMP-induced activation of TLR2 may induce T_H_2-dominated chronic inflammatory responses as well as induction of SMAD3 and STAT3 with subsequent recurrence of suppressive immune cells (T_reg_, M2) and cytokines (TGF- β) leading to the progression of fibrosis. Kim and colleagues further revealed reduced production of IL-27 by epithelial cells and enhanced IL-17 production from immune cells in TLR2^−/−^ mice compared to WT mice [163]. The authors argued that BLM-mediated activation of TLR2 on AECs triggers the production of IL-27 and chemokines thereby inhibiting the production of IL17 and recruitment of repair-promoting inflammatory cells.

Altogether these findings suggest that TLR4 is needed for limiting the massive inflammation and associated damage induced by acute intratracheal BLM administration, whereas subsequent activation of TLR2 signaling may promote disease progression by altering the effector class of the immune response. The observed exacerbation of both, BLM and WTI-induced pulmonary fibrosis in TLR2^−/−^/TLR4^−/−^ mice highlights that multiple and time-dependent signals seem to be required to shape an optimal immune response in order to orchestrate inflammation, resolution and regeneration. But if the damage response is too excessive, or the resulting immune response is deregulated, persistent damage associated with chronic inflammation can result in the progression of pulmonary fibrosis. In this context, endogenous TLR ligands such as HA fragments may even cooperate with endogenous activators of the inflammasome such as uric acid to boost inflammatory responses in the injured lung [164].

The differences between TLR2^−/−^ and TLR4^−/−^ mice in the response to intratracheal BLM and WTI point to a distinct contribution of TLR2 and TLR4 to disease pathogenesis in the acute and chronic models of DNA damage-induced lung injury that may be linked to the differences in the type of the primary damage, the extent of the initial damage, or its persistence (ability to repair or replace primary damaged epithelial versus endothelial cells). Furthermore, the fact that BLM can act as a direct TLR2 ligand might explain why signaling via TLR2 triggers a more pronounced initial damage and toxic inflammation only in the BLM model but not in the WTI model [162]. On the other hand, the failure of TLR4^−/−^ mice to develop a more pronounced fibrosis upon WTI might be a hint that TLR4-mediated beneficial effects e.g. on inflammatory resolution may be compensated by protective signaling from other TLR receptors. Thus, only the loss of both, TLR2-dependent and TLR4-dependent immunoregulatory effects seems to be sufficient to disturb the delicate balance between inflammation and repair required for fibrosis development in irradiated lungs.

Studies in other injury models and investigations in patients with idiopathic pulmonary fibrosis (IPF) suggest a contribution of further TLR such as TLR3, TLR7 or TLR9 in the pathogenesis of pulmonary fibrosis [157, 165–169]. It will therefore be important to study the specific beneficial and adverse effects of the diverse TLRs and of their cross-talk with other immune regulatory molecules in more detail if we aim to modulate danger signaling for improving disease outcome.

### Role of MyD88 signaling in DNA damage-induced pneumopathy

The adaptor protein MyD88 is a key regulator of immunity. Due to its critical role in signaling of all TLR except from TLR3 a genetic deficiency of MyD88 in mice results in an altered immune response to danger and stress induced by pathogens or damage, including DNA damage. Thus, further insight about the role of TLR signaling in DNA damage-induced pneumopathy has been achieved by studies in MyD88 knockout mice (MyD88^−/−^). However, it has to be taken into account that MyD88 is not only an intermediary between activated TLR (except TLR3) and immune activation but also participates in the transmission of signals from IL1R/IL18R. It is therefore not surprising that multiple reports highlight a role of MyD88 in lung injury induced by WTI or BLM; but again, the obtained results differ depending of the models used.

Brickey and colleagues analyzed the role of MyD88 deficiency in MyD88^−/−^ mice applying a single high dose WTI with 14 Gy. They found that MyD88^−/−^ mice were characterized by increased sensitivity to radiation-induced cell death in the lung [170], thereby corroborating the findings obtained in TLR2/TLR4 double knockout mice [161]. Increased sensitivity of MyD88^−/−^ mice to the adverse effects of WTI was characterized by enhanced interstitial immune cell infiltration during the pneumonitic phase at 4–6 weeks post-irradiation, more sites of focal damage at later stages (18–27 weeks post-irradiation) as well as enhanced collagen deposition when compared to irradiated WT mice [170]. Interestingly, MyD88^−/−^ mice had increased levels of T_H_2 like cytokines in the lungs during the fibrotic phase. These findings demonstrate that MyD88 has a protective role in radiation-induced adverse late effects in the lung and seems to be critical for the regulation of inflammation and regeneration after WTI, presumably by transmitting signals from TLR2/TLR4.

Pulmonary fibrosis was also exacerbated in MyD88^−/−^ mice in response to intratracheal BLM administration [158]. Here, lack of MyD88 signaling caused reduced transepithelial migration of neutrophils, enhanced epithelial cell apoptosis, and decreased survival of mice after lung injury compared to WT mice and thus also corroborated the findings obtained in TLR2^−/−^/TLR4^−/−^ mice. The authors proposed that endogenous matrix molecules, like hyaluronan and the interplay with the TLR2-TLR4-MyD88 pathway have crucial protective functions in the defense against sterile lung injury [158]. While Brickey and coworkers found enhanced interstitial infiltrates with lymphocytes and CD11b^+^ cells in MyD88^−/−^ mice in response to WTI, Jiang and colleagues measured increased levels of polymorphnuclear neutrophils in the bronchoalveolar lavage fluid (BALF) of MyD88^−/−^ mice exposed to intratracheal BLM administration. Thus, though both studies focused on different immune cells they both demonstrate that the TLR2-TLR4-MyD88 pathway is crucial for the defense against sterile lung injury in response to WTI and intratracheal BLM administration.

In contrast, MyD88 signaling exerted disease-promoting effects in the model of intranasal BLM application [171]. Loss of MyD88 reduced the influx of immune cells as well as lung inflammation and the development of pulmonary fibrosis. The authors argued that the controversial findings might be due to the different dose and application route of BLM used by Jiang et al. [158] and their own study [171]: They speculated that intranasal application of BLM might induce weaker damage responses in the lung, because liquids are less homogeneously distributed in the respiratory tract and potentially also spread to the gastric tract [172]. A similar argumentation was used in a study comparing intratracheal, intranasal, and oropharyngeal BLM administration in rats [173]. The gut is an organ with particularly high tolerance to PAMPs/DAMPs so that swallowing of part of the BLM dose may further limit the initial danger signal generated by intranasal BLM administration in the respiratory tract [173]. Importantly, the disease-promoting effects of MyD88 in the model of intranasal BLM administration seemed to be independent of TLR2/4 signaling but rather linked to the function of MyD88 in IL1R1 signaling since IL1R^−/−^ but not TLR2^−/−^/TLR4^−/−^ mice were also protected from BLM-induced fibrosis in this model [171]. The disease-promoting effects of MyD88 might therefore be due to synergistic effects on IL-1β signaling via TLR-mediated induction and inflammasome-mediated activation of IL-1β in this model.

As discussed in the TLR section, the investigations in the MyD88 model support the assumption that the time-course and the extent of the initial damage, for example due to different types, localization or extent of damage induction (WTI or intratracheal versus intranasal BLM application) will shape the damage response and the resulting inflammation and thereby largely influence the pathogenic process.

### Effectors of DNA damage-induced pneumopathy

#### Role of hyaluronan (HA)

The glycocalyx of endothelial and epithelial cells recently emerged as an important participant in both, inflammation and immunomodulation upon injury and may thus be of use as disease biomarkers or novel target (s) for therapeutic interventions aimed at immune modulation [174]. One of the major extracellular matrix proteins, the glucosaminoglycan HA, is biologically inert or exerts anti-inflammatory effects as long as it is present in its physiological high molecular weight (HMW) form [174]. However, if HA becomes oxidized, fragmented or both during tissue injury, low molecular weight (LMW) HA fragments can act as DAMPs and stimulate inflammation and repair, presumably by interacting with TLR2 and TLR4 [175–179]. In contrast, there is increasing evidence that treatment with HMW HA or reduction of HA degradation may be suited to reduce inflammatory processes induced by LMW HA [180–183].

In the lung, HA is mainly located in the peribronchial and interalveolar/perialveolar tissue and has important functions in normal lung homeostasis and pulmonary disease as described in detail elsewhere [183–185]. HA impacts tissue injury, inflammation and repair by regulating inflammatory cell recruitment, release of inflammatory cytokines, and cell migration. These various regulatory activities are controlled by the regulation of HA-synthesis and HA-degradation via hyaluronan synthases and hyaluronidases, respectively. Furthermore, expression of the various HA binding proteins (HABPs), such as HABP2, the HA receptors CD44 and *receptor for HA-mediated motility* (RHAMM), as well as TLR2 and TLR4 also participate in controlling the actions of HA [185–187]. It has been suggested that interaction of RHAMM with HA may be critical for inflammatory cell recruitment to the injured lung [186]. Furthermore, HA, TLR2, RHAMM and TGFβ1 were required for surfactant protein A-stimulated macrophage chemotaxis [188]. Instead, HA and CD44 seem to be important for the pro-fibrotic phenotype of recruited fibroblasts as genetic deficiency or pharmacologic inhibition of CD44 reduced the aggressive phenotype and pulmonary fibrosis, though pulmonary inflammation was increased [189, 190].

Expression of HMW HA at the surface of epithelial cells can provide protection against tissue damage potentially by binding to macrophage CD44 [191] or signaling through TLR2 and TLR4 [187]. In contrast LMW HA fragments generated in response to oxidative stress, injury or hyaluronidase-activation impact lung homeostasis and participate in inflammation-associated disease processes such as asthma, chronic obstructive pulmonary disease (COPD) as well as pulmonary hypertension and fibrosis [158, 189, 192, 193]. LMW HA also signals through TLR2, TLR4 and the HA receptor CD44 to stimulate inflammatory gene expression [185]. Thus, CD44 and TLRs mediate cell responses to HA of different molecular weight with opposing outcomes.

An interesting observation was that HA/TLR2 and HA/TLR4 interactions alter sensitivity to lung inflammation and fibrosis upon lung injury. It is thought that impaired clearance of damage-induced HA fragments may promote chronic inflammation. In this context, signaling of HA fragments via TLR2 and TLR4 was shown to regulate the inflammatory response to BLM-induced lung injury and promote recovery and repair, at least in the acute BLM model [158, 187]. In line with this assumption, acute intratracheal BLM administration caused a reduced trans-epithelial immune cell migration but enhanced epithelial cell apoptosis, chronic inflammation and reduced survival of TLR2 ^−/−^/TLR4^−/−^ and MyD88^−/−^ mice [158].

Interestingly, HA fragments isolated from individuals with lung injury triggered inflammatory responses in response to acute lung injury via TLR4, TLR2 and MyD88 signaling cascades, presumably by stimulating macrophage cytokine production [158]. Instead, expression of HMW HA on the surface of lung epithelial cells protected against acute lung injury amongst others by promoting TLR-dependent basal activation of NF-κB and rescue from apoptosis [158]. The concept that signaling of HMW HA via TLRs may initiate repair-promoting inflammatory processes in the lung that maintain epithelial cell integrity upon acute injury has recently been supported by a study showing that HMW HA and TLR4 are both expressed on AECII cells and cooperate in promoting the renewal of these cells thereby preventing severe BLM-induced fibrosis in mice [187]. It appears that the effects of HA on inflammatory processes largely depend on both, HA-related factors such as size and location as well as cell-specific factors such as expression of HA synthases, HA receptors and the respective signaling pathways [179]. We therefore speculate that these differences might at least partially explain tissue-specific and damage-specific importance of TLR2, TLR4 and MyD88 in the outcome of DNA damage-induced injury.

#### Role of purinergic signaling

Another important immune checkpoint in the focus of current research is the purinergic signaling pathway (ATP to adenosine conversion). The dual roles of extracellular adenosine, during acute and chronic inflammatory disease stages in various organs have been summarized elsewhere [194, 195]. Herein a causal link between chronic accumulation of adenosine, increased matrix deposition and fibrosis development had been revealed in the skin and the lungs of mice with genetic deficiency of adenosine deaminase (ADA) [196–198].

We recently demonstrated that activation of CD73 signaling and subsequent chronic accumulation of immunoregulatory adenosine in the irradiated lungs are important drivers of pulmonary fibrosis in response to WTI with 15Gy. Genetic loss of CD73 or pharmacological inhibition with a CD73-antibody or by degradation of adenosine with pegylated-adenosine deaminase (PEG-ADA) significantly reduced levels of pro-fibrotic mediators and attenuated the extent of pulmonary fibrosis. Loss of CD73 also reduced apoptosis in lung epithelial cells during the acute inflammatory phase [12]. Thus, chronic activation of CD73 and progressive accumulation of adenosine in the irradiated lung exert pro-fibrotic actions during chronic disease stages of radiation-induced pneumopathy. Furthermore, fibrosis development in WT mice was associated with a prominent up-regulation of hyaluronic acid synthase 2 (HAS2) and a time-dependent deposition of HA. Instead, loss of CD73 inhibited the radiation-induced deposition of excess HA and prevented the accumulation of alternatively activated macrophages in pre-fibrotic clusters [13]. Interestingly, this was associated with an altered regulation of components of the HA system e.g. delayed up-regulation of HAS2 as well as specific up-regulation of the HA receptor CD44 and of HAS3 that mainly synthesizes LMW HA during the fibrotic phase [199, 200]. Though expression of HAS2 reached similar levels in irradiated WT and CD73^−/−^ mice during the fibrotic phase the failure of irradiated CD73^−/−^ mice to chronically accumulate adenosine may abrogate the pro-fibrotic signaling loop involving adenosine, HAS2, and TGFβ thereby limiting fibrosis development. Instead, up-regulation of the HAS3 during the fibrotic phase may help the CD73^−/−^ mice to limit fibrosis development, e.g. by activating ECM-degrading enzymes [201]. We therefore speculate that pro-fibrotic signaling in response to WTI involves a cross-talk between CD73/adenosine signaling, HA and other pro-fibrotic mediators.

In contrast to our findings, loss of CD73 increased the sensitivity of C57BL/6 mice to lung inflammation and fibrosis induced by intratracheal BLM administration demonstrating CD73-dependent adenosine accumulation has protective effects in this acute damage model. BLM treatment induced a massive infiltration of immune cells and inflammation in the lungs of CD73^−/−^ mice as well as increased production of pro-inflammatory and fibrotic mediators compared to WT mice. Moreover, CD73^−/−^ mice developed more pronounced pulmonary fibrosis and displayed a higher mortality than WT mice. Of note, restoration of CD73 function in CD73^−/−^ mice by intranasal administration of additional AMPase enhanced adenosine levels and reduced inflammation and fibrosis development in response to intratracheal BLM [202]. Thus CD73 and extracellular adenosine exert beneficial effects in the acute intratracheal model of BLM-induced lung injury and fibrosis, presumably by activating anti-inflammatory pathways.

However, chronic intraperitoneal application of BLM induced a chronic accumulation of adenosine and exacerbated lung inflammation and fibrosis, thereby revealing pronounced differences between the acute model of intratracheal BLM administration and the chronic model of intraperitoneal BLM administration [79, 198, 203]. The pathogenic role of adenosine in chronic pulmonary lung disease had mostly been linked to ADORA2B and myeloid cells, at least in murine models [79, 204, 205]. Moreover, an association between ADORA2B and chronic pulmonary disease has also been described in patients [206]. Similar to our findings in the WTI model, multiple reports point to a multifaceted cross-talk between the immunoregulatory effects of adenosine and HA with impact on fibrosis development [206–208]: While LMW HA was shown to down-regulate the anti-inflammatory ADORA2A receptor, stimulation of ADORA2A inhibited LMW HA-induced expression of pro-fibrotic cytokines such as TNFα, macrophage inflammatory protein (MIP)-1α and MIP-2 but synergized with LMW HA in inducing IL-12. These interactions may provide an explanation why ADORA2A^−/−^ mice are more sensitive to BLM-induced inflammation, accumulation of HA and histologic damage [207].

Altogether these findings indicate that adenosine has beneficial effects in the acute model of BLM-induced lung injury but adverse effects in the chronic models of DNA-damage induced pneumopathy. We speculate that under conditions of chronic adenosine accumulation, adenosine, HA and TLR2/TLR4/MyD88 participate in a fibrosis-promoting signaling network that may be driven at least in part by a TGFβ-mediated increase in HAS2 [209, 210], CD73-induced adenosine generation [12], as well as effector cells expressing HA synthases, HA-degrading enzymes, and TLR2/4 receptors as described above.

However, it has also to be taken into account that purinergic signaling has dual functions in immune regulation. Extracellular release of ATP from damaged or stressed cells contributes to pro-inflammatory responses via P2Y/P2X receptor signaling. Herein, degradation of immunostimulatory ATP into immunosuppressive or immuneregulatory adenosine serves as a negative-feedback mechanism able to counteract overwhelming inflammation. Interestingly, there are some hints for a cross-talk between signaling cascades initiated by TLR ligands and ATP: for example the release of mature IL1β seems to rely on the cooperation of two events, namely the NF-κB-mediated induction of pro-IL1β, for example downstream of TLR activation, as well as caspase-1-mediated cleavage as a result of an active NLRP3 inflammasome and activation of the ATP receptor P2X7 [211]. However the requirement for ATP and P2X7 activation seems to depend on the cell type and the expression and/or activation of TLR2 versus TLR4: while TLR2 agonists required pannexin-1 to release endogenous ATP and P2X_7_ receptor activation to stimulate IL-1β release from monocytes (at least in the absence of exogenous ATP), LPS triggered IL-1β independently of pannexin-1 and ATP-mediated P2X_7_ activation [212]. This requirement of ATP for the activation of the inflammasome downstream of TLR2 but not TLR4 signaling might provide another explanation for the specific disease-promoting effects of the BLM receptor TLR2 in the model of BLM model that is not observed in the chronic WTI model. Furthermore, these differences might explain why deficiency of TLR2 has beneficial effects and loss of TLR4 adverse effects in the acute model of intratracheal BLM administration as this will impact the balance between TLR-mediated and inflammasome-mediated immune signaling.

### The role of cytokine signaling in DNA damage-induced pneumopathy

Besides investigations about damage signaling via TLR2, TLR4, and MyD88, researchers also focused on the role of specific pro- or anti-inflammatory cytokines in the pathogenesis of DNA-damage induced pneumopathy. The recognition of PAMPs and DAMPs by PRR triggers an immune response that results in maturation, activation and secretion of diverse cytokines and chemokines in target cells. Depending on the interacting molecular pattern and the cell type several pro-inflammatory responses e.g. TNF-α, IL-6, IL-1, IL-12, IFNγ can be triggered [113]. In contrast to early pro-inflammatory responses is the late induction of anti-inflammatory and immunosuppressive cytokines e.g. IL-4, IL-10, IL-13 in the altered microenvironment during pneumopathy [213–215].

#### Lack of IL-6 signaling

One important cytokine in driving pro-inflammatory responses is IL-6. IL-6 contributes to pulmonary inflammation and a dysregulation of this cytokine has an impact on the severity of multiple respiratory diseases [216–219]. Therefore, researchers used IL6-deficient (IL-6^−/−^) mice to study the contribution of this cytokine to WTI-induced and BLM-induced pneumopathy.

Exposure of mice to WTI with 10 Gy (X-rays) induced up-regulated expression of markers for inflammation (CD44, 1 h post irradiation) and apoptosis (Bak, 1d post irradiation) in the lungs of C57BL/6 WT mice but not in IL-6^−/−^ mice during the acute phase. In contrast, CD44 expression was increased in IL-6^−/−^ mice at day 3 post-irradiation suggesting that IL-6 impacts the early inflammatory phase after thoracic irradiation [220]. But when using 10 Gy WTI with carbon ions (C-Ions), another radiation quality, and focusing on the fibrotic phase the same group showed that IL-6^−/−^ mice had reduced levels of pulmonary fibrosis compared to irradiated C57BL/6 WT mice at 24 weeks post-irradiation pointing pro-fibrotic actions of IL6 during the chronic pathological state [221].

Further work revealed that IL-6 has also disease-promoting effects in the acute model of BLM-induced lung fibrosis: Deficiency of IL-6 provided a partial protection from fibrosis induced by intratracheal BLM administration that was associated with reduced numbers inflammatory cells at very early time points (4 h hours after BLM administration) [222]. Though the numbers of inflammatory cells reached similar levels as BLM-treated WT mice at day 7 post-treatment IL-6^−/−^ mice showed lower levels of TGF-β and CCL3 as well as less pronounced collagen content and fibrosis than WT mice at day 21 after BLM treatment [222]. In a related study blockade of IL-6 inhibited proliferation of fibroblasts, suggesting that the action of IL-6 on fibroblasts may provide a more important contribution to the pro-fibrotic effects of IL-6 during the chronic phase of lung injury than its action on immune cells [223].

The biphasic induction of IL-6 upon intratracheal BLM administration has recently been corroborated in another study where IL-6 levels peaked at days 0.5–3 and at days 8–10 after BLM treatment, respectively [218]. Surprisingly, pharmacologic inhibition of IL-6 by administration of a neutralizing antibody during the early phase increased apoptosis in AECII cells, neutrophil infiltration to the lung and fibrosis development, whereas IL-6 inhibition during the second phase ameliorated pulmonary fibrosis [218]. Again, IL-6 seems to have a dual role during BLM-induced lung injury: while it exerts an anti-fibrotic role during the early stage presumably by protecting IL-6 producing AECII in an autocrine/paracrine manner, IL-6 produced by macrophages and fibroblasts might promote pulmonary fibrosis [218].

The anti-inflammatory and anti-apoptotic effects of IL-6 deficiency observed in the WTI model and the BLM model during the early phase using IL6^−/−^ mice differs from the pro-apoptotic effects of pharmacological inhibition of IL-6 in the acute BLM model using an IL-6 neutralizing antibody. This might be at least partially due to the distinct disease markers, methods and time points used. However, it has also to be taken into account that pharmacologic inhibition allows for an acute, time-restricted inhibition of a certain pro-fibrotic molecule in a well-defined treatment schedule. These effects may differ from data obtained in knockout mice where the respective signaling molecule is chronically absent and may trigger adaptive changes in the tissue of interest, the immune system, or both, as already suggested by others [224].

#### Lack of IL-4 signaling

The cytokine IL-4 is an important mediator of T_H_2 differentiation and plays a key role in anti-inflammatory responses. Furthermore IL-4 has been described as a pro-fibrotic cytokine due to its capacity to induce an enhanced matrix deposition in fibroblasts [225]. Therefore, researchers also investigated the impact of IL-4 deficiency in animal models of radiation-induced and BLM-induced pneumopathy.

Genetic deficiency of IL-4 did not protect IL-4^−/−^ mice from pulmonary fibrosis induced by WTI with 12.5 Gy measured at 26 week post-irradiation [226]. The equal sensitivity of WT mice and IL-4^−/−^ mice pulmonary fibrosis upon WTI argues against a critical role of IL4 in this process that might be explained be potential compensatory effects of the related cytokine IL-13: IL-13 also binds to receptors shared with IL-4, thereby mediating pro-fibrotic actions [227, 228].

IL-4 deficiency did also not alter the sensitivity of mice to pulmonary fibrosis induced by intratracheal administration of BLM (0.06 mg, 1 mg = 1 U) when investigated at 14 days post BLM-treatment [83, 229]. Instead, transgenic IL-4 overexpression protected mice from fibrosis development, suggesting that enhanced IL-4 levels might improve the tolerance of the lung tissue to injury induced by intratracheal BLM administration [83]. It is widely accepted that IL-4 modulates the expression of inflammatory cytokines like IL-α1, IL-1β TNF-α, IFN-γ and others [229]. An overexpression of IL-4 might thus counteract the excessive inflammatory damage induced by intratracheal BLM treatment under certain conditions, thereby limiting fibrosis development. This is supported by findings obtained in a model of acute lung injury induced by immune complexes where the additional instillation of IL-4 into the lungs reduced TNF-α, ICAM-1, myeloperoxidase and neutrophil levels compared to untreated mice [230].

Instead, Huaux and co-workers revealed that a lower dose of intratracheal BLM (0.05 U) induced an excessive T_H_1 response and a higher and earlier mortality in IL-4^−/−^ mice compared to WT animals [214]. Thus, IL-4 may well exert important protective effects in the lung tissue exposed to intratracheal BLM administration. However, when using 0.02 U BLM pulmonary fibrosis evaluated at day 28 turned out to be less severe in IL-4 deficient mice compared to WT mice. Thus, IL-4 may also exert time-dependent dual effects in the lung tissue by modulating immune cell responses and thus BLM-induced inflammation at early time points thereby limiting lung injury and fibrosis, but promote fibrosis development at later stages when fibroblasts play a major role [214].

We assume that the extent of the initial damage will dictate the dominant effect of IL-4 in the injured lung: under conditions of severe initial damage, loss of IL-4 will boost an uncontrolled early T_H_1 response leading to chronic damage and massive fibrosis development at later time points. In contrast, under conditions of a less pronounced damage the dominant effect of IL-4 relates to fibroblast behavior during the fibrotic stage so that loss of IL-4 will limit pathogenic fibroblast activities and fibrosis development.

These intriguing observations from the WTI and BLM models indicate that cytokines such as IL-4 and IL-6 may have dual roles during the pathogenesis of DNA damage-induced pneumopathy, namely, limiting overwhelming pro-inflammatory responses during the early phase and promoting pro-fibrotic actions of macrophages, fibroblasts or both during the fibrotic stages. Furthermore, the findings obtained in the models of cytokine deficiency corroborate the conclusions made in the models of TLR/MyD88 signaling and the other immune checkpoints discussed above, that the severity of the initial insult will orchestrate the extent and direction of inflammation, resolution and repair.

### Role of the cells from the adaptive immune system

The mechanisms of damage-induced immune activation follow a common principle where professional innate immune cells and non-professional resident cells sense danger, produce a cocktail of mediators and activate lymphocyte responses to activate specific effector responses. So far, the potential contribution of B-lymphocytes and T lymphocytes to DNA-damage induced pneumonitis and fibrosis has only been investigated in single studies using either thorax irradiation or BLM and distinct mouse strains. We summarized the current knowledge on the role of lymphocytes in radiotherapy-induced adverse late effects in the lung in a recent review [142]. Therefore, we will highlight only some important observations that underline the suggested differences in the role of cells from the adaptive immune system between the pathogenesis of the BLM and the radiation models in the following paragraphs.

#### Studies using inbred mouse strains with defects in the adaptive immune system

One common model to study the role of B/T cells in pathogenic processes is the use of *recombination-activating gene* (RAG)-1 deficient (RAG1^−/−^) or RAG2^−/−^ mice, that lack mature T and B lymphocytes [231, 232]. In our hands RAG2^−/−^mice turned out to be more sensitive to WTI with 15 Gy than C57BL/6 WT (WT) mice as they developed more prominent fibrosis already at week 24 when compared to WT mice [233]. These findings suggest that the tissue protective effects of lymphocytes infiltrating the irradiated lung tissue predominate so that overall mature lymphocytes contribute to the control of radiation-induced adverse late effects in the lung. The earlier onset of fibrosis in the RAG2^−/−^mice might be linked to a disturbed balance between innate and adaptive immune responses, resulting in a more pronounced tissue-destruction [233].

Unfortunately, the sensitivity of RAG1^−/−^ or RAG2^−/−^ mice to BLM-induced pulmonary fibrosis has not yet been explored. However, in the related model using instillation of the irritating chemical compound fluorescein isothiocyanate (FITC) the lack of functional T cells in RAG2^−/−^ mice had no effect on the sensitivity to FITC-induced fibrosis [234] pointing to potential differences in the role of lymphocytes in radiation-induced and chemotherapy-induced pulmonary fibrosis.

Instead the role of lymphocytes in BLM induced fibrosis has been investigated in SCID mice and athymic *Nude* mice that have a deteriorated thymus resulting in reduced numbers of T cells, with some controversial findings: Intratracheal administration of BLM (3 U/kg) to C57BL/6 SCID mice led to strongly decreased IL-2 levels compared to C57BL/6 WT mice but had no impact on the levels of TNF-α, IL-12 and IFN-γ and the development of pulmonary fibrosis. Therefore, the authors concluded that non-lymphoid immune responses are sufficient for the development of BLM-induced pulmonary fibrosis whereas lymphocytes may not be important [235]. Similarly, Szapiel et al. observed comparable alveolitis and fibrosis in control and athymic *Nude* mice upon repeated intraperitoneal injections of BLM (low 8x20U/kg, medium 8x50U/kg and high dose 8x125U/kg) supporting that the adaptive immune system is not critical role for the pathogenesis in BLM-induced pneumopathy [236].

In contrast, athymic *Nude* mice were partially protected from the development of pulmonary fibrosis upon intratracheal BLM (3 U/kg) administration [237]. Here the authors concluded that lymphocyte responses may well play a role in regulating the fibrogenic response in the lung at least after intratracheal BLM instillation [237]. Thus, SCID mice and athymic *Nud*e mice showed different outcomes in the acute model of intratracheal BLM administration, although the same dose of BLM was administered. This might at least partially be linked to the distinct genetic background of the mice, since SCID mice have a C57BL/6 background whereas athymic *Nude* mice have an albino background. Nevertheless, such partial protective effects were not observed in athymic *Nude* mice exposed to intraperitoneal BLM, leading to the assumption that the route and dose of BLM administration impact on damage-induced immunological responses, the severity of inflammation and subsequent fibrosis development.

Of note the defect in the adaptive immune system in *Nude* mice is less severe than in SCID mice as *Nude* mice still have functional B cells. It is therefore tempting to speculate that the absence of potential disease-promoting T cell subsets and/or the presence of B cells in *Nude* mice may provide some protection, at least in the model of intratracheal BLM administration. However other findings argue against a protective role of B cells in pulmonary fibrosis: in one study overexpression of the B cell receptor CD19 correlated with enhanced pulmonary fibrosis compared to WT mice upon intratracheal BLM administration. Furthermore CD19^−/−^ mice showed reduced fibrosis and improved survival compared to WT mice, suggesting a potential harmful role for B cells in the development of pulmonary fibrosis upon BLM treatment [238].

The conflicting findings on the role of lymphocytes in BLM-induced pulmonary fibrosis have been summarized and discussed earlier elsewhere [224]. We agree with the assumption of the authors that the route and schedule of BLM administration seems to be critical to treatment outcome and might dictate the role of cells from the adaptive immune system in regulating the tissue response to BLM-induced damage. Furthermore, we also agree that the controversial findings about the role of T and B cells in BLM-induced lung fibrosis obtained in SCID, *Nude* and CD19^−/−^ mice might at least partially be due to the reported ability of genetically modified murine strains to adapt to their respective immune defects by rewiring “inflammatory and repair mechanisms in a context-dependent manner” [224]. The different inbred mouse strains may thus compensate their defects in a distinct fashion resulting in different damage responses.

So far, the role of lymphocytes in WTI-induced or BLM-induced pulmonary fibrosis has never been directly compared in the same genetically modified mouse strain. Therefore, one can only speculate about potential differences in the role of lymphocytes in both models. To our view lymphocytes play a dual role in the WTI-model: During the latent phase and the pneumonitic phase T_H_1 responses may contribute to tissue inflammation, whereas CD4^+^ regulatory T cells (T_reg_) seem to counteract excessive inflammation [239, 240]; instead during the chronic phase T_H_2 responses and T_reg_ might promote fibrosis development [12, 240]. The earlier onset of WTI-induced fibrosis in RAG-2^−/−^ mice when compared to WT mice might be attributed to an imbalance between the innate and adaptive immune systems in the damage response: As summarized recently, innate lymphoid cells (ILC), such as ILC2, and diverse cells of the innate immune system are suspected to influence the damage response during the acute phase. The absence of mature T/B cells might lead to disturbed control of innate immune cells resulting in distinct changes in the lung environment with impact on the repair capacity of the lung tissue and fibrosis development [142].

### Final remarks

WTI and intratracheal BLM instillation are two common models to study the pathogenesis of DNA-damage induced pneumonitis and fibrosis with the aim to identify predictive disease biomarkers, to test additional toxicities of new combination strategies, and/or to develop effective mechanisms-based strategies for the prevention or treatment of adverse late effects of RT or RCT in the lung. The data summarized and discussed here highlight that both models display substantial differences in the role of various molecules with impact on damage-induced immune activation such as TLR2/4, MyD88, CD73/adenosine, hyaluronan, or cells from the adaptive immune system and their mediators. Thus, the two models differ in the complex response to DNA damage initiated by administration of BLM or WTI. Thus, it will be crucial to use the radiation models instead of the BLM model if we are to define reliable biomarkers for radiation-induced pneumopathy or to test the efficacy of combined RCT protocols or RT in combination with radioprotective drugs or mitigating agents in preclinical models: the differences in the contribution of immune cells and immune regulatory molecules observed between the WTI and BLM models may become critical when testing the effect of immune checkpoint inhibitors or immunotherapies alone or in combination therapies.

One important difference relates to the time-response associated with the type of damage as we compare a highly acute damage response upon intratracheal BLM-treatment to a chronic damage response in the WTI model and to a lesser extent in the model of chronic intraperitoneal BLM administration. Furthermore, the primary target of DNA damage (epithelial cell versus endothelial cell) seems to be another important determinant of the tissue response to DNA damage, as we observe obvious differences in the immune activation and fibrosis development between the intratracheal, the intranasal and the intraperitoneal BLM application route. Finally, the extent of damage represents another critical determinant of the inflammatory response: If the damage is manageable, the inflammation will be in balance with the resolution phase, normal repair and subsequent regeneration. However, if the initial damage is too severe, the inflammatory phase will be more excessive, leading to uncontrolled inflammation with subsequent chronic damage and fibrosis development. Finally, if the initial insult triggers a late cell demise and chronic inflammation, (radiation-induced) fibrosis may also develop. Consequently, targeting immune checkpoints or other immunoregulatory molecules will have different effects in these settings as highlighted above for TLR2 and TLR4-signaling, the ATP/adenosine immune checkpoint and the cytokines IL-4 and IL-6, respectively.

Cells with an anti-inflammatory capacity and their mediators that are needed to control inflammation in the acute phase can contribute to immune deviation and a pro-fibrotic microenvironment under conditions of persistent damage and vice-versa. Similarly, induction of senescence in resident or locally recruited cells may have either beneficial effects by inducing protective immune responses or disrupt tissue homeostasis and promote chronic inflammation to drive the progression of radiation–induced pneumopathy. In this context, increased induction or decreased clearance of senescent cells can support therapy-induced accumulation and persistence of senescent cells thereby promoting chronic inflammation and disease pathogenesis [241].

Besides irradiation, ROS and reactive nitrogen species (RNS) are potent stressors that can induce senescence [29] and promote pneumonitis and fibrosis [242, 243]. Moreover, though inflammatory mediators are usually considered to be part of the SASP, Inflammation and inflammatory cytokines can also participate in the induction of senescence and SASP in an autocrine or paracrine manner [32]. For example, IL-6 acts via IL-6R in an autocrine loop to promote oncogene-induced senescence at least in vitro [32, 244]. The same holds true for IL-8 and its receptors CXCR1 and CXCR2 [244–246]. Finally, DAMPs, e.g. extracellular HMGB1, have been described to enhance secretion of SASP components and to promote senescence-associated inflammation via TLR2 and TLR4 [247]. It has been shown that DAMPs, cytokines and their receptors are up-regulated in the lung in response to DNA damage [218, 248, 249] either during the pneumonitic phase, the fibrotic phase, or both. Therefore it is tempting to speculate that DAMPs and inflammatory cytokines/mediators impact senescence induction and the pulmonary SASP phenotype upon DNA damage. Vice-versa radiation-induced senescence and SASP might be important mediators and/or amplifiers of radiation-induced normal tissue injury, inflammation and bystander effects, which in turn enhance inflammation and stress-induced senescence [31]. Thus, senescence could either be the cause or the consequence of persistent inflammation upon DNA damage [29]. However the respective DAMPs remain to be identified.

We therefore assume that simple inhibition of inflammatory pathways may not be successful in improving treatment outcome for patients with chronic disease since the inflammation-associated damage may already have occurred and inflammatory states may be needed as to initiate repair processes. Instead we propose that time-dependent balancing of certain pathological aspects of tissue inflammation may be suited to attenuate radiation-induced chronic lung disease [12, 13]. Furthermore, adoptive transfer of mesenchymal stem cells (MSC) can be used to attenuate radiation-induced vascular damage and fibrosis development [19, 250], e.g. by increasing in tissue levels of superoxide dismutase (SOD) and improving the stress resistance of the lung tissue to chronic damage triggered by ROS [250]. A similar protective effect can be achieved by treating the lung tissue with SOD-mimetic drugs, as already shown by others [250–252]. While therapeutic approaches with antioxidants like vitamin E or C to treat fibrosis did not fulfil the expectations (nicely summarized by [243]), targeting or reducing a ROS/RNS-enriched environment by the above-mentioned treatment with SOD-mimetics (e.g. EUK-207, EUK-189) or Nox/Nrf2 inhibitors or even adoptive MSC transfer seems be more effective, at least in animal experiments [19, 253, 254]. Particularly the adoptive transfer of MSC seems to be a promising novel treatment option as it targets multiple pathological alterations such as radiation-induced loss of SOD, immune deviation and vascular remodeling presumably by paracrine signaling [19, 250]. Finally, targeting ROS-induced senescence or apoptosis-resistant senescent cells might be another promising novel approach to limit DNA damage-induced normal tissue toxicity in the lung [25, 27, 255]. Nevertheless, there is still a lack of knowledge about the interplay of ROS, inflammation, senescence and fibrosis development and further studies are needed to clarify the therapeutic potential of targeting DNA damage-induced pneumopathy, particularly pulmonary fibrosis.

Increasing tissue tolerance to injury by exposure to a distinct type of damage [256] turns out to be another promising concept to prevent or treat the adverse effects of microbial infection, inflammation, or DNA damaging treatment, respectively. This has been demonstrated for example by the protection against severe sepsis trough treatment with DNA damaging anthracyclines [257, 258]. In support of the concept that tolerance of the lung to microbial threats can be increased by exposure to damage by chemotherapy, heat or ionizing radiation low doses of X-rays have been used historically in the 1930th to treat pneumonia in an attempt to suppress inflammatory responses, facilitate disease resolution and reverse clinical symptoms (for a review see [259]). Furthermore, though exposure of healthy normal tissue to high dose irradiation is known to trigger adverse local inflammatory reactions such as mucositis and pneumonitis, or even autoimmune responses [260–263], exposure to low dose local or whole body/total lymphoid irradiation is effective in promoting survival of cells in damaged tissues [264] and even attenuates benign chronic inflammatory or autoimmune disease [265–271].

Vice-versa challenging an irradiated host with compounds mimicking microbial infection such as flagellin-derived TLR5 ligands is well suited to protect sensitive tissues including the lungs from the adverse effects of chemotherapy and RT in preclinical models [272–275]. Interestingly, TLR5 ligands promote natural killer cell, dendritic cell and CD8^+^ T cell-mediated immunity in various settings suggesting a potential role of such responses in radioprotection [276–278]. In a related approach, treatment with the TLR-9 ligand CpG-oligodeoxynucleotide (CpG-ODN) that is known as a potent vaccine adjuvant for anticancer therapy, attenuated radiation-induced lung fibrosis [279, 280] amongst others by up-regulating the expression of TLR9, and increasing Type-1 immunity [280]. Finally, intranasal vaccination with vaccinia particles was shown to attenuate BLM-induced fibrosis by inhibiting the generation of M2 macrophages and the recruitment of fibrocytes [281]. Interestingly, vaccination with vaccinia enhanced survival of C57BL/6 WT mice but not RAG1^−/−^ mice or IFN-γ-deficient mice exposed to BLM pointing again to a role of lymphocytes and Th1 immune responses [281]. Herein, targeting the HA signaling network may be another approach to increase tissue tolerance to radiation or BLM treatment [182, 187].

Increasing tissue tolerance to ionizing radiation by promoting TLR-induced activation of NF-κB might thus be well suited to protect radiosensitive tissues with little repair capacity such as the lung from the adverse effects of IR presumably by promoting survival of epithelial cells and balancing chronic immune deviation. However, deficiency of TLR3 provided mice with substantial resistance towards the lethal radiation-induced gastrointestinal syndrome suggesting that the inhibition of TLR3-associated TRIF-dependent signaling may be protective [282].

In the present review, we aimed to focus on similarities and differences in the role of danger signals and their cross-talk with the innate and adaptive immune systems in the response of the lung tissue to DNA damage induced by BLM-treatment or exposure to ionizing radiation. Furthermore, we aimed to highlight novel approaches exploiting the respective knowledge for the identification of new therapeutic targets. These novel targets include for example danger signals, death resistance, progressive senescence, or immune deviation [12, 24, 26, 27]. Of course, further aspects of radiation-induced lung toxicity research and promising ways of radiation mitigation and protection have been described in preclinical models, for example the use of the free radical scavenger Amifostine, inhibition of the renin-angiotensin system (RAS) with angiotensin converting enzyme (ACE) inhibitors [283–285], or agents modulating the DNA damage response. But since the use of such mitigators has been summarized in detail elsewhere [286, 287] we did not include a detailed discussion of these approaches in the present review.

Taken together, further studies in the radiation models are required to complete our understanding about the complex interplay between radiation-induced damage on the tissue level, the resulting damage responses of the various resident cells, senescence and programmed cell death, danger signals, immune activation and further environmental changes that are specific to the irradiated lung and promote chronic lung disease after thoracic irradiation. Furthermore, investigations with tissue-specific knockout mice and depletion of certain immune cell populations will be required to address the importance of the respective signaling molecules, specific cell types, or both. A better understanding of the complex signaling networks and cell-cell interactions is required if we aim to exploit the damage response, tolerance mechanisms, or both to prevent or treat radiation-induced lung disease. These shall include investigations in models of (hypo) fractionated irradiation as well as models using particle therapy whereas data obtained in the murine model of BLM-induced lung injury should be considered with care.
